# Evaluation of the White Test for the Intraoperative Detection of Bile Leakage

**DOI:** 10.1155/2012/425435

**Published:** 2012-04-03

**Authors:** Kawin Leelawat, Kittipong Chaiyabutr, Somboon Subwongcharoen, Sa-ad Treepongkaruna

**Affiliations:** ^1^Department of Surgery, Rajavithi Hospital, Bangkok 10400, Thailand; ^2^College of Medicine, Rangsit University, Bangkok 10400, Thailand

## Abstract

We assess whether the White test is better than the conventional bile leakage test for the intraoperative detection of bile leakage in hepatectomized patients. This study included 30 patients who received elective liver resection. Both the conventional bile leakage test (injecting an isotonic sodium chloride solution through the cystic duct) and the White test (injecting a fat emulsion solution through the cystic duct) were carried out in the same patients. The detection of bile leakage was compared between the conventional method and the White test. A bile leak was demonstrated in 8 patients (26.7%) by the conventional method and in 19 patients (63.3%) by the White test. In addition, the White test detected a significantly higher number of bile leakage sites compared with the conventional method (Wilcoxon signed-rank test; *P* < 0.001). The White test is better than the conventional test for the intraoperative detection of bile leakage. Based on our study, we recommend that surgeons investigating bile leakage sites during liver resections should use the White test instead of the conventional bile leakage test.

## 1. Introduction

Postoperative bile leakage is one of the most common causes of sepsis and liver failure after liver surgery [1, 2]. Previous studies demonstrated that the incidence of postoperative bile leakage after liver surgery ranges from 3 to 27% [2–5]. The conventional intraoperative bile leakage test, which involves injecting an isotonic sodium chloride solution through the cystic duct, has been used to detect leakage from the transected liver surface [6]. One of the major problems in using the conventional bile leakage test is that the isotonic sodium chloride solution is a transparent solution. Therefore, it is difficult to detect the point of bile leakage. A previous randomized study indicated that there is no advantage to using the isotonic sodium chloride solution for the bile leakage test during liver resection [6]. Recently, intraoperative application of the White test has been demonstrated to reduce the incidence of postoperative bile leakage [7, 8]. In this technique, bile leakage sites on the transected liver surface are identified by injecting a fat emulsion solution through the cystic duct. The previous prospective observational studies suggested that the fat emulsion solution used in the White test is easily recognized, innocuous to the tissues, and can be easily removed without misleading tissue staining [7, 8]. Therefore, we executed a prospective study to assess whether the White test is better than the conventional bile leakage test for the intraoperative detection of bile leakage.

## 2. Materials and Methods

### 2.1. Patients

This study included patients who were scheduled for elective liver resection at Rajavithi Hospital from August 2010 to August 2011. Informed consent was obtained from the patients, and this study was approved by the Rajavithi Hospital ethics committee.

### 2.2. Interventions

Liver tissue was transected using a harmonic scalpel with intermittent occlusion of hepatic inflow [9, 10]. After complete liver resection, bleeding from the liver surface was stopped, and the visible points of bile leakage were secured with interrupted sutures placed with an atraumatic needle. Both the conventional bile leakage test and the White test were then performed in the same patients. To perform the tests, a catheter was inserted through the cystic duct into the common bile duct. For the conventional bile leakage test, 10–20 mL of isotonic sodium chloride solution was injected via the catheter while manually occluding the distal common bile duct. The transected liver surface was then inspected by two surgeons for the leakage of any isotonic sodium chloride solution. After marking any sites of bile leakage, the residual isotonic sodium chloride solution was removed by syringe aspiration via the catheter. The White test was then performed during the same surgery. To perform the White test, 10–20 mL of a 5% sterile fat emulsion was slowly injected while manually occluding the distal common bile duct. The presence of the white fluid was then assessed at bile leakage sites on the transected liver surface. The number of bile leakage sites found with the conventional method was then compared with the number found with the White test. After finishing the tests, the detected bile leakages were closed with interrupted sutures (5–0 or 6–0 polydioxanone sutures). Drainage of the operative field was performed with a silicone drain connected to a closed drainage system (Jackson-Pratt drain). Postoperative bile leakage was defined as bilirubin concentration in the drain fluid at least 3 times the serum bilirubin concentration on or after postoperative day 3 or at the need for radiologic or operative intervention resulting from biliary collections or bile peritonitis [11].

### 2.3. Statistical Analysis

Sample size was calculated on the basis of an expected difference of 2.0 between the mean number of bile leakage sites detected by the conventional method and the White test, with an estimated standard deviation of 2.0. Using a significance level of 0.05 (two sided) and a power of 0.95, at least 18 hepatectomized patients were required for the study. The continuous variables were expressed as the mean ± SD. A paired *t*-test or Wilcoxon signed-rank test was used to compare the number of bile leakage sites identified by the conventional method and the White test in each patient. Differences at *P* < 0.05 were considered statistically significant.

## 3. Results

A total of 30 hepatectomized patients were enrolled. The subjects studied included 4 patients with intrahepatic duct stones, 3 patients with benign tumors, 5 patients with liver metastasis from colorectal cancer, 8 patients with cholangiocarcinoma, and 10 patients with hepatocellular carcinoma. All of these patients were in the Child-Pugh A classification. The clinical characteristics of the patients are shown in Table 1.

**Intraoperative Identification of Bile Leakage**

To determine whether the conventional bile leakage test or the White test is superior for the intraoperative identification of bile leakage, both tests were performed on all patients after completing the liver resection. A bile leak was demonstrated in 8 patients (26.7%) by the conventional method and in 19 patients (63.3%) by the White test. In addition, the White test identified a significantly higher number of bile leakage sites than the conventional method (Wilcoxon signed-rank test; *P* < 0.001).

We divided these patients into 2 groups according to liver resection type [16]: 12 patients received minor liver resections (<3 liver segments were resected), and 18 patients received major liver resections (>3 liver segments were resected). The results showed that the White test identified a significantly higher number of bile leakage sites than the conventional method in both groups (Wilcoxon signed-rank test; *P* = 0.016 for minor liver resections group and *P* = 0.002 for major liver resections group) (Table 2).

The postoperative course for all patients was uneventful. There was neither operative mortality nor serious morbidity. There are 2 cases of transient bile leakage occurred and spontaneously resolved within 3 wk without any intervention (grade A bile leakage [11]).

## 4. Discussion

Previous randomized trials have investigated the role of the conventional bile leakage test during liver resection [6]. Bile leakage was demonstrated and repaired by the conventional bile leakage test in 41% of patients. However, the incidence of postoperative bile leakage did not significantly differ between the group receiving the conventional bile leakage test and the control group. Recent studies demonstrated that bile leakage test with fluorescent dye solution could detect bile leakage that could not be identified by a conventional bile leak test [12, 13]. Therefore, we hypothesize that the transparent sodium chloride solution used in the conventional bile leakage test is the major problem in detecting bile leakage sites. However, specialist equipment and expertise for performing the bile leakage tests with fluorescent dye solution were not available at every hospital.

The White test uses fat emulsion, which is normally used for parenteral nutrition for localization of bile leakage [14]. In this study, we demonstrated that the White test is better than the conventional test for the intraoperative detection of bile leakage. In the subgroup analysis, we also demonstrated that the White test is superior to the conventional test in both minor and major liver resections groups. A previous prospective study included 74 patients receiving no bile leakage test as a control group and 63 patients undergoing the White test as the study group [7]. Postoperative bile leakage was found in 22.9% patients in the control group and in 5.3% patients in the White test group, respectively (*P* < 0.01). Our study result agreed with the previous studies in that a bile leakage test cannot definitely prevent postoperative bile leakage because all of the biliary stumps on the transected liver surface cannot be identified by this technique [15]. In our study, postoperative bile leakage was found in 2 patients (6.6%). Both received major liver resections (one patient received right trisegmentectomy for hepatocellular carcinoma and the other received left trisegmentectomy for intrahepatic cholangiocarcinoma). Previous study indicated that high-risk procedure (hepatectomies in which the cut surface exposed the major Glisson's sheath and included the hepatic hilum) is the independent risk factor for development of bile leakage [1]. One of our bile leakage patients has liver cirrhosis. However, previous study suggested that liver cirrhosis was not associated with the increase incidence of bile leakage [15]. The association of liver parenchyma disease and the incidence of bile leakage should be further evaluated.

Taken together, we recommend that surgeons investigating the presence of bile leakage sites during liver resections should utilize the White test instead of the conventional bile leakage test. However, large randomized trials of the White test during major and minor liver resections should be performed before routinely using the test for liver resection procedures.

## Figures and Tables

**Table 1:** Patient characteristics and operative variables.

Variable	Patients (*n* = 30)
Age (mean ± SD; Yr)	56.0 ± 10.21
Sex (male : female)	16 : 14
Type of liver resection (%)	
Lobectomy	18 (60)
Segmentectomy	9 (30)
Subsegmentectomy	3 (10)
Blood loss (mean ± SD; mL)	880 ± 240
Operation time (mean ± SD; min)	350 ± 242

**Table 2:** Comparison between the conventional test and the White test for the detection of bile leakage.

Type of liver resection	Number of bile leakage sites (mean ± SD)		*P* value*
	Conventional test	White test	
Minor (12 cases)	0.4 ± 0.79	1.9 ± 1.82	0.016
Major (18 cases)	0.3 ± 0.46	1.4 ± 1.69	0.002
Total (30 cases)	0.3 ± 0.60	1.6 ± 1.70	<0.001

*Wilcoxon signed-rank test.
